# Polymorphisms in estrogen-metabolizing and estrogen receptor genes and the risk of developing breast cancer among a cohort of women with benign breast disease

**DOI:** 10.1186/1471-2407-6-173

**Published:** 2006-06-29

**Authors:** Lisa Gallicchio, Sonja I Berndt, Meghan A McSorley, Craig J Newschaffer, Lucy W Thuita, Pedram Argani, Sandra C Hoffman, Kathy J Helzlsouer

**Affiliations:** 1 Department of Epidemiology, Johns Hopkins Bloomberg School of Public Health, Baltimore, Maryland, USA; 2 Prevention and Research Center, The Weinberg Center for Women's Health and Medicine, Mercy Medical Center, Baltimore, Maryland, USA; 3 Department of Pathology, Johns Hopkins School of Medicine, Baltimore, Maryland, USA

## Abstract

**Background:**

A cohort study was conducted to examine the role of genetic polymorphisms in three estrogen metabolizing enzymes (*COMT*, *CYP1A1*, *CYP1B1*) and the two estrogen receptors (*ESR1*, *ESR2*) in the progression of benign breast disease (BBD) to breast cancer.

**Methods:**

Among participants in an ongoing cohort study, 1438 Caucasian women had a breast biopsy for BBD and were successfully genotyped for at least one of the polymorphisms examined in this study. Genotypes were determined using DNA extracted from blood specimens collected in 1989. Incident cases of breast cancer occurring subsequent to BBD diagnosis up to 2003 were identified through cancer registries.

**Results:**

Among all participants, the ESR2 *5772G allele was associated with a significant decrease in the risk of breast cancer among women with BBD (Odds Ratio (OR) 0.38; 95% Confidence Interval (CI) 0.15, 0.96). Compared to the reference wild-type genotypes, marginally significant associations with the development of breast cancer were observed between carriers of the variant *ESR1 – 104062T *allele (OR 0.70, 95% CI 0.45, 1.09), the variant ESR2 *38A allele (OR 1.40; 95% CI 0.88, 2.25), and the variant *CYP1B1 453Ser *allele (OR 1.48, 95% CI 0.95, 2.32).

**Conclusion:**

The results indicate that specific polymorphisms in the *CYP1B1, ESR1*, and *ESR2 *genes may play a role in progression of BBD to breast cancer among Caucasian women. Although additional studies are needed to confirm or refute our findings, these results suggest that genetic markers may aid in the identification of women who are at risk for progression of BBD to cancer.

## Background

Biopsy-proven benign breast disease (BBD) is a well-defined risk factor for developing invasive breast cancer [1,2]. However, little is known about which women with BBD progress to have a subsequent *in situ *or invasive cancer. A woman's level of endogenous estrogen may be important in the progression of BBD to invasive breast cancer. Abundant evidence suggests that estrogen is an important factor in the development of breast cancer among average-risk women. This evidence includes findings from recent prospective studies that showed significantly increased levels of serum estrogen in women who developed breast cancer compared to women who did not develop breast cancer [3-7]. In addition, numerous studies have reported that risk factors associated with prolonged estrogen exposure, such as early age at menarche, nulliparity, and older age at first birth, increase a woman's risk of developing breast cancer (reviewed by Mitrunen and Hirvonen [8]).

Because of the importance of estrogen in the development of breast cancer among average-risk women, it is plausible that polymorphisms in estrogen metabolizing genes may be associated with the risk of developing breast cancer among women with BBD. Estrogens undergo oxidative metabolism by several cytochrome P450 (CYPs) enzymes [9]; the major metabolic pathways are 2-hydroxylation, which is primarily catalyzed by *CYP1A1*, and 4-hydroxylation, which is dominated by *CYP1B1 *[10]. The hydroxylated estrogens are then either converted to semiquinones or quinones, which can produce DNA adducts and lead to oxidative damage to lipids and DNA, or inactivated by catechol O-methyltransferase (*COMT*) [10-13]. Within the genes encoding for *CYP1A1*, *CYP1B1*, and *COMT*, a number of polymorphisms have been identified, some which have been shown in laboratory studies to be functional (8). The associations of some of these polymorphisms with the risk of developing breast cancer among average risk women have been studied, and most of the findings have been inconsistent (reviewed in Mitrunen and Hirvonen [8]). No study has examined the associations of polymorphisms in *CYP1A1*, *CYP1B1*, and *COMT *with breast cancer risk among women with BBD.

While the estrogen metabolizing enzymes may influence the levels of circulating endogenous estrogens and, therefore, contribute to breast cancer risk, the estrogen receptors determine, in part, the action of estrogens on the mammary gland. Estrogen acts on target tissues by binding to the estrogen receptors, which exist in two forms, ER-α or *ESR1 *and ER-β or *ESR2 *[14,15]. Like the estrogen metabolizing enzymes, a number of polymorphisms in both ER genes have been identified [16], and it has been hypothesized that these polymorphisms may increase or decrease a woman's breast cancer risk depending on the functional consequence of the polymorphism. Several studies have investigated the associations between polymorphisms in the *ESR1 *and *ESR2 *genes and the risk of breast cancer among average-risk women and have reported mixed results [16-29]. To our knowledge, however, no study has examined the associations of polymorphisms in *ESR1 *and *ESR2 *with breast cancer risk among women with BBD.

The aim of the present study was to analyze the associations between genetic polymorphisms in three estrogen metabolizing enzymes (*COMT*, *CYP1A1*, *CYP1B1*) and the two estrogen receptors (*ESR1*, *ESR2*) and the risk of developing breast cancer among women with BBD. To address this aim, data from a cohort of women with BBD from Washington County, Maryland enrolled in the CLUE-II study were analyzed.

## Methods

### CLUE II cohort

In 1989, a campaign named CLUE II ("*Campaign against Cancer and Heart Disease*") was conducted in Washington County, Maryland to establish a cohort with associated blood samples [30]. At baseline, participants signed a consent form, completed a brief medical and exposure history and a food frequency questionnaire, and donated a blood sample. The blood sample was collected in heparinzed tubes and centrifuged to obtain separation of the buffy coat which was then aliquotted and stored at -70°C. DNA was subsequently extracted from the buffy coat using standard phenol extraction [31].

A total of 25,081 persons (10,456 men and 14,625 women), about 30 percent of the Washington County residents, participated in the study. In addition to the information collected from this cohort at baseline in 1989, follow-up data were obtained from questionnaires administered in 1996 and approximately every 2 years thereafter. The Institutional Review Board at the Johns Hopkins Bloomberg School of Public Health approved this study.

### BBD cohort

The BBD cohort is a sub-set of CLUE II. Details of the selection of CLUE II participants included in the BBD cohort are described elsewhere [32]. Briefly, women who responded on the 1996 CLUE II follow-up questionnaire that they had undergone a "breast biopsy or lumpectomy" were considered for inclusion into the BBD cohort. Women were included in the BBD cohort if either they were found to have BBD on their first located pathology report or a pathology report was not found but a breast biopsy was reported prior to or in the absence of a subsequent breast cancer diagnosis. Women who were diagnosed with breast cancer at their first biopsy, as reported by the participant or as determined using the pathology report search, were excluded from this analysis. In total, of the 1683 women who reported that they had a breast biopsy, 1467 women (87.2%) met the criteria above and were included in the BBD cohort. Among these 1467 women, pathology report data were collected for 362 (24.7%). Seventy percent of these pathology reports indicated a diagnosis of non-proliferative BBD, 26% indicated a diagnosis of proliferative disease without atypia, and 4% reported a diagnosis of proliferative disease with atypia.

For this analysis, 17 of the 1467 women were excluded because DNA could not be obtained from their stored blood samples and polymorphisms could not be genotyped. Further, all non-Caucasians (n = 12) were excluded from the analysis because previous studies have shown that race is an important confounder or effect modifier in investigations of polymorphisms and disease. These exclusions accounted from less than 1% of the BBD cohort. After applying these exclusions, the final analytic cohort included 1438 Caucasian women who were successfully genotyped for at least one of the genetic polymorphisms investigated in this study.

### Outcome measurement

Incident cases of *in situ *or invasive breast cancer (International Classification of Diseases, Ninth Revision, code 233, 174) were identified through linkage of the cohort participants with the Washington County Cancer Registry and, since 1992, with the Maryland State Cancer Registry. The Washington County Cancer Registry identifies cancer cases from discharge records and pathology reports from Washington County Hospital, which is the only hospital in the county, and from death certificates. Since 1992, all hospitals cancer diagnostic laboratories, and radiation therapy centers in Maryland have been required to report incident cancer cases to the Maryland Cancer Registry, and currently, the Maryland Cancer Registry is certified as being more than 95% complete by the North American Association of Central Cancer Registries. While it is possible that cases of *in situ *or invasive breast cancer may have been missed due to migration out of the state after 1996, all women in the BBD cohort had at least 7 years of follow-up data (1989 to 1996). Further, approximately 80% of the BBD cohort responded to the 2000 follow-up questionnaire, indicating that the vast majority of the cohort remained under active surveillance in 2000.

Active case surveillance for the BBD cohort ended on April 28, 2003. In total, among all of the women included in the BBD cohort, 15 incident cases of *in situ *and 76 cases of invasive breast cancer were identified after the diagnosis of BBD.

### Genotyping

Single nucleotide polymorphisms (SNPs) analyzed in the present study were selected as part of a larger study investigating the broad impact of genetic variation in candidate genes and their interactions with environmental exposures on cancer incidence and survival. For the larger study, polymorphisms that were suggested to alter function, encoded for a nonsynonymous amino acid change, or were located within the 5' or 3' untranslated region (UTR) of the gene and thus could potentially alter mRNA stability were chosen. Descriptions and dbSNP identifiers of the polymorphisms selected are shown on Table 1. Genotyping of the chosen SNPs in *COMT, CYP1B1, CYP1A1, ESR1 *and *ESR2 *was conducted at Applied Biosystems (ABI) and Celera Laboratories. All polymorphisms were genotyped using TaqMan technology (ABI, Foster City, CA, USA). The genotyping success rates of the polymorphisms selected ranged from 91.8% to 97.6%, with the exception of the *CYP1B1 Arg48Gly *polymorphism for which the genotyping success rate was 84.2%. The 84.2% genotyping success rate for *CYP1B1 Arg48Gly *polymorphism was due to a problem with the laboratory assay that was not resolved during the study period.

### Statistical analysis

The outcome variable examined in these analyses was the development of *in situ *or invasive breast cancer. Unconditional logistic regression was used to estimate age-adjusted odds ratios (ORs) and 95% confidence intervals (95% CIs) for the associations between the polymorphisms the development of breast cancer. Because previous studies indicate that the associations between several of the analyzed polymorphisms and the development of cancer may differ according to body mass index, smoking status, and alcohol intake, we conducted exploratory analyses to examine the associations between all of the polymorphisms and the development of breast cancer among strata of these variables (BMI: <25 kg/m^2^, ≥25 kg/m^2^; smoking status: ever, never; alcohol intake: current, no current use). Further, these analyses were conducted for all participants and for pre- and post-menopausal participants separately. Because the number of events among the pre-menopausal women was low (n = 24) and ORs for some of the genotypes could not be calculated, only the analyses for all participants and the post-menopausal subgroup are reported. As an additional exploratory analysis, logic regression [33] was employed using the software package R 2.0.1 to examine whether any interactions between the genotypes existed.

To address the issue of multiple testing in this study, p-values for the associations between single nucleotide polymorphisms and breast cancer risk were adjusted for the false discovery rate using the method proposed by Benjamini and Hochberg [34] in the software package R 2.0.1.

Unless otherwise specified, analyses were performed using SAS Version 8.2 (Cary, NC). A p-value of less than 0.05 was considered to be statistically significant.

## Results

Characteristics of the analytic cohort are reported in Table 2. The mean age of the participants was 53.5 years (standard deviation 12.1), and the majority of the women had at least a high school degree (82.2%). Approximately 88% of the women had been pregnant at least once in their lifetime, 16.2% were current smokers, and 28.8% were current drinkers. Among the 1438 women in this sample, 91 (6.3%) developed invasive or *in situ *breast cancer after an initial breast biopsy for BBD.

All of the polymorphisms examined were in Hardy-Weinberg equilibrium among women not developing breast cancer, with the exception of the *CYP1B1 Ile462Val *polymorphism (p-value = 0.02). Although statistically significant, the observed genotype counts for the *CYP1B1 Ile462Val *polymorphism were similar to the expected genotype counts under Hardy-Weinberg equilibrium (observed/expected: AA-1233/1229.5; AG-80/86.9; GG-5/1.5). All *ESR2 *polymorphisms were in strong linkage disequilibrium (D' > 90). Among the *ESR1 *polymorphisms, the *Ser10Ser *and *Ala87Ala *polymorphisms were strongly linked (D' > 90).

The associations between polymorphisms in the estrogen metabolizing enzymes and the risk of developing invasive breast cancer among women with BBD are shown in Table 3. Among all participants and post-menopausal participants only, carriers of at least one *CYP1B1 453Ser *allele had a borderline statistically significant increase in the risk of developing breast cancer compared to women carrying the referent *Asn/Asn *genotype (all participants: OR 1.48, 95% CI 0.95, 2.32; post-menopausal participants: OR 1.61; 95% CI 0.94, 2.74). The other polymorphisms in the estrogen metabolizing enzymes were not associated with breast cancer risk among women with BBD in this study. Results were not substantially altered by BMI, alcohol intake or smoking status (results not shown). No significant interactions between the genotypes were observed. To note, after adjustment for multiple testing, none of the associations between polymorphisms in the estrogen metabolizing enzymes and the risk of developing invasive breast cancer were statistically significant.

The associations between polymorphisms in *ESR1 *and *ESR2 *and the risk of developing invasive breast cancer among women with BBD are shown in Tables 4 and 5. A marginally significant decrease in the risk of developing breast cancer was observed among carriers of the *ESR1 – 104062T *allele compared to carriers of the referent genotype (all participants: OR 0.70, 95% CI 0.45, 1.09). Among all participants, the ESR2 *5772G allele was associated with a statistically significant decrease in the risk of breast cancer among women with BBD (OR 0.38; 95% CI 0.15, 0.96). A marginally significant increase in the risk of developing cancer was observed among women carrying at least one ESR2 *38A allele compared to the women homozygous for the GG genotype (all participants: OR 1.40; 95% CI 0.88, 2.25). OR estimates of the ESR2 *5772G and the ESR2 *38A polymorphisms were attenuated after adjustment for the presence of the other polymorphism (ESR2 *5772G OR 0.42; 95% CI 0.17, 1.05; ESR2 *38 OR 1.27; 95% CI 0.78, 2.04). Other *ESR1 *and *ESR2 *polymorphisms examined were not associated with breast cancer risk among women with BBD. Results did not differ substantially by BMI, alcohol intake or smoking status (results not shown). No significant interactions between the *ESR1 *and *ESR2 *genotypes were observed. After adjustment for multiple testing, the associations between the *ESR1 *and *ESR2 *polymorphisms and breast cancer described above were not statistically significant.

## Discussion

In this population-based cohort of women with BBD, there was evidence of an increased risk of breast cancer among women who carried the *CYP1B1 453Ser *allele compared to women who carried two copies of the *CYP1B1 453Asn *allele. None of the previously published studies on the *CYP1B1 Asn453Ser *polymorphism and breast cancer conducted among average-risk women reported an increase in risk of breast cancer associated with this polymorphism [35-38], including a recent meta-analysis that reported an OR of 0.91 (95% CI 0.79, 1.04) associated with the heterozygous *Asn/Ser *genotype and an OR of 0.85 (95% CI 0.54, 1.34) associated with the homozygous *Ser/Ser *genotype [39]. However, functionality studies demonstrate that the *CYP1B1 *polymorphic variant is associated with both higher catalytic activity in converting estrogen to 4-hydroxy estrogens and greater levels of DNA damage [39-41]. This mechanism may be more relevant for progression among the subgroup of women with BBD who progress to breast cancer, thus it is reasonable to examine this association among women biopsy proven BBD.

A marginally significant positive association was observed for the ESR2 *38G>A. This finding is not consistent with results of published studies among either average-risk or high-risk women. Maguire et al [26] reported no increase in breast cancer risk associated with two copies of the variant *38A allele among average-risk (OR 0.81; 95% CI 0.50, 1.31) or high risk (OR 1.00; 95% CI 0.58, 1.71). In addition, Gold et al [16] observed that haplotypes containing the common ESR2 *38G allele were associated with an increased risk of breast cancer among Ashkenazi Jews. These differences in findings may be due to the different populations under study; Maguire et al [26] examined *ESR2 *polymorphisms among patients in Sweden and Gold et al (16) investigated *ESR2 *and breast cancer among the Ashkenazi Jews. Neither investigated the association of *ESR2 *polymorphisms among women diagnosed with BBD.

In addition, results from this study suggest that women with BBD who carry the ESR2 *5772A>G polymorphism or the *ESR1 – 104062C>T *polymorphism are at decreased risk of developing breast cancer. To our knowledge, neither of these genotypes has been examined in women with BBD or among average risk women. The regions surrounding each polymorphism are well conserved with little recombination, therefore, it likely that, if the associations are real, the observed associations are due to linkage disequilibrium with another functional polymorphism in the region. No information on functionality is know for either ESR2 *5772A>G or *ESR1 – 104062C>T*.

There was no evidence of an association between the other polymorphisms examined in this study, including those in *COMT *and *CYP1A1*, and breast cancer risk. Although no published study has investigated the *COMT Val158Met *and *CYP1A1 Ile462Val *polymorphisms in the progression of BBD to invasive breast cancer, previous studies of average-risk women have reported discrepant results regarding the association between the *COMT Val158Met *and the *CYP1A1 Ile462Val *polymorphisms and the risk of breast cancer [8,42]. Both polymorphisms have been observed to be functional; the *COMT 158Met *allele is associated with three- to four-fold decreased activity compared to the wildtype allele [43] and the *CYP1A1 462Val *allele is consistent with higher enzyme activity [44]. Therefore, an association between these polymorphisms and breast cancer is biologically plausible and support the findings of studies that show an association between the polymorphisms and breast cancer risk. It may be, however, that both polymorphisms only modify the association between endogenous estrogens and the risk of breast cancer. Incorporating data on circulating estrogen concentrations may clarify the importance of carrying a specific genotype. Alternatively, it may also be that the effect of any single polymorphisms on the risk of breast cancer is small and that it is necessary to look at combinations of variants in the hormone metabolism pathway.

While this study provides novel information regarding the role of polymorphisms in estrogen metabolizing enzymes and the estrogen receptor genes in the progression of BBD to breast cancer, this study has several limitations inherent to its design. As reported in a previously published investigation on this cohort of women [32], pathology reports were not able to be obtained for all women who reported that they had undergone a surgical biopsy. This was due both to difficulties in obtaining permission for pathology review for some participants as well as to an inability to obtain records of biopsies done more than 15 to 20 years ago. Since concordance of finding a pathology report and report of biopsy among women granting permission for pathology review who reported that they had undergone a biopsy after 1980 was high (92.5%), all women who reported a breast biopsy were included in the analyses. However, this may have led to the inclusion of a small number of women without BBD. Further, because data on the type of BBD were not collected for all participants, the analyses conducted in this study were not stratified by type of BBD, and, therefore, the ORs reported may underestimate (or overestimate) the true associations among women with certain types of BBD.

In addition, though the base cohort is relatively large, the number of cases of breast cancer occurring limited the power of the study to make firm conclusions about the relationships between polymorphisms in *COMT*, *CYP1B1*, *CYP1A1*, *ESR1 *and *ESR2 *and the risk of breast cancer in Caucasian women with BBD. We had limited power to detect small associations and, therefore, were also limited in our ability to correct for multiple testing. As stated in the results section, the associations between the polymorphisms and the risk of developing breast cancer were not statistically significant after correction for multiple testing and this may have been due, in part, to the small sample size. However, while we may be able to rule out large changes in risk of clinical and public health consequence associated with certain genotypes, we still observed, with the small number of events, statistically significant changes in the risk of breast cancer in this cohort. Taken together with functional considerations of these polymorphisms, it is important to follow-up on these observations to determine if we can better understand and perhaps intervene for those women with BBD who are most at risk to progress to cancer.

## Conclusion

The findings of this study indicate that specific polymorphisms in the *CYP1B1, ESR1*, and *ESR2 *genes may play a role in progression of BBD to breast cancer among Caucasian women. Although additional studies are needed to confirm or refute our findings, these results suggest that genetic markers may aid in the identification of women who are at risk for progression of BBD to cancer.

## Abbreviations

BBD Benign breast disease

COMT Catechol O-methyltransferase

CYPs Cytochrome P450 enzymes

ESR1 Estrogen receptor-alpha

ESR2 Estrogen receptor-beta

SNP Single nucleotide polymorphism

## Competing interests

The author(s) declare that they have no competing interests.

## Authors' contributions

LG contributed to the statistical analysis, interpretation of the data, and the writing of the manuscript. SIB provided expertise on the more advanced analyses of the genetic data and contributed to the interpretation of the data. MAM, LWT, and SAH contributed to the data collection and statistical analysis. PA conducted reviews of the pathology report data and pathology specimens. CJN and KJN contributed to the conception and design of the study and the interpretation of the data.

## Pre-publication history

The pre-publication history for this paper can be accessed here:



## Figures and Tables

**Table 1:** Description of single nucleotide polymorphisms examined in the progression of BBD to breast cancer

Gene	dbSNP rs #	snp description	Amino Acid Substitution
*COMT*	4680	Ex3-12G>A	Val158Met
*CYP1A1*	1048943	Ex7+131A>G	Ile462Val
*CYP1B1*	1800440	Ex3+315A>G	Asn453Ser
*CYP1B1*	10012	Ex2+143C>G	Arg48Gly
*CYP1B1*	1056836	Ex3+251G>C	Val432Leu
*ESR1*	1801132	Ex4-122G>C	Pro325Pro
*ESR1*	2077647	Ex1+392T>C	Ser10Ser
*ESR1*	746432	Ex1-192G>C	Ala87Ala
*ESR1*	2273206	IVS6+52G>T	
*ESR1*	851982	-104062C>T	
*ESR1*	2228480	Ex8+229G>A	Thr594Thr
*ESR2*	4986938	38 bp 3' of STP C>T	
*ESR2*	928554	5659 bp 3' of STP G>A	
*ESR2*	none	5696 bp 3' of STP A>G	
*ESR2*	8018687	5772 bp 3' of STP A>G	

**Table 2:** Characteristics of the study cohort (n = 1438)

Characteristic	n	%
Race		
Caucasian	1438	100.0
Age, years^a^		
<50	533	37.1
50 to 60	411	28.6
>60	494	34.3
*Missing*	*0*	0.0
Education, years^a^		
less than a high school degree	259	18.0
high school degree	714	49.7
some college	463	32.2
*Missing*	*2*	0.1
Menopausal status^a^		
Pre-menopausal	377	26.2
Peri-menopausal	35	2.4
Post-menopausal	995	69.2
*Missing*	31	2.2
Family history of breast cancer^a^		
No	715	49.7
Yes	275	19.1
*Missing*	*448*	31.2
Ever been pregnant (%)^b^		
No	176	12.2
Yes	1255	87.3
*Missing*	*7*	0.5
Age at first birth (years, %)^b^		
Nulliparous	176	12.2
20	347	24.1
20 to 24	539	37.5
≥25	339	23.6
*Missing*	*37*	2.6
Age at menarche (years, %)^b^		
<12	268	18.6
12 to 13	763	53.1
>13	386	26.8
*Missing*	*21*	1.5
Height, inches^a^		
<62	411	28.6
62–64	419	29.1
>64	608	42.3
*Missing*	*0*	0.0
Weight, pounds^a^		
<130	354	24.6
130–<145	318	22.1
145–<165	377	26.2
≥165	387	26.9
*Missing*	*2*	0.1
BMI, kg/m^2, a^		
<24.9	744	51.7
25.0 – 29.9	433	30.1
≥30	259	18.0
*Missing*	*2*	0.1
Smoking status^a^		
Never	839	58.3
Former	366	25.5
Current	232	16.1
*Missing*	*1*	0.1
Current alcohol drinker^a^		
No	814	56.6
Yes	413	28.7
*Missing*	*211*	14.7

^a ^data from 1989 questionnaire

^b ^data from 1996 questionnaire

**Table 3:** Associations between estrogen metabolizing polymorphisms and breast cancer risk in the BBD cohort

		Cancer		No Cancer			
	dbSNP rs#	(n = 91)		(n = 1347)		All Participants	Post-menopausal Only
		n	%	n	%	Age- Adjusted OR (95% CI)	Age- Adjusted OR (95% CI)
	
*COMT Val158Met*	4680						
GG		16	17.6	272	20.2	1.00	1.00
GA		41	45.1	608	45.1	1.15 (0.63, 2.08)	0.89 (0.45, 1.77)
AA		24	26.4	371	27.5	1.10 (0.57, 2.11)	0.90 (0.43, 1.90)
							
GA/AA		65	71.4	979	72.7	1.13 (0.64, 1.98)	0.89 (0.47, 1.70)
Missing		10	11.0	96	7.1		
							
*CYP1A1 Ile462Val*	1048943						
AA		78	85.7	1233	91.5	1.00	1.00
AG		7	7.7	80	5.9	1.38 (0.62, 3.10)	1.30 (0.45, 3.74)
GG		1	1.1	5	0.4	3.33 (0.38, 28.99)	8.33 (0.74, 93.62)
*p-for-trend*						0.20	0.20
							
AG/GG		8	8.8	85	6.3	1.49 (0.70, 3.19)	1.56 (0.60, 4.08)
Missing		5	5.5	29	2.2		
							
*CYP1B1 Asn453Ser*	1800440						
AA		51	56.0	866	64.3	1.00	1.00
AG		31	34.1	358	26.6	1.45 (0.91, 2.31)	1.51 (0.87, 2.65)
GG		4	4.4	37	2.7	1.77 (0.61, 5.16)	2.44 (0.81, 7.36)
*p-for-trend*						0.08	0.05
							
AG/GG		35	38.5	395	29.3	1.48 (0.95, 2.32)	1.61 (0.94, 2.74)
Missing		5	5.5	86	6.4		
							
*CYP1B1 Arg48Gly*	10012						
CC		39	42.9	600	44.5	1.00	1.00
CG		33	36.3	460	34.1	1.10 (0.68, 1.78)	0.95 (0.53, 1.69)
GG		3	3.3	76	5.6	0.60 (0.18, 2.00)	0.25 (0.03, 1.88)
							
CG/GG		36	39.6	536	39.8	1.02 (0.65, 1.64)	0.84 (0.48, 1.50)
Missing		16	17.6	211	15.7		
							
*CYP1B1 Val432Leu*	1056836						
CC		25	27.5	365	27.1	1.00	1.00
CG		39	42.9	653	48.5	0.88 (0.53, 1.48)	1.04 (0.56, 1.98)
GG		17	18.7	270	20.0	0.93 (0.49, 1.76)	0.93 (0.41, 2.09)
							
CG/GG		56	61.5	923	68.5	0.90 (0.55, 1.46)	1.01 (0.56, 1.85)
Missing		10	11.0	59	4.4		

**Table 4:** Associations between estrogen receptor-alpha (*ESR1*) polymorphisms and breast cancer risk in the BBD cohort

		Cancer		No Cancer			
	dbSNP rs#	(n = 91)		(n = 1347)		All Participants	Post-menopausal Only
		n	%	n	%	Age- Adjusted OR (95% CI)	Age-Adjusted OR (95% CI)
	
*ESR1 Pro325Pro*	1801132						
CC		52	57.1	794	58.9	1.00	1.00
CG		31	34.1	440	32.7	1.07 (0.67, 1.69)	1.06 (0.61, 1.87)
GG		7	7.7	64	4.8	1.65 (0.72, 3.78)	2.03 (0.81, 5.10)
							
CG/GG		38	41.8	504	37.4	1.14 (0.74, 1.76)	1.19 (0.70, 2.01)
Missing		1	1.1	49	3.6		
							
*ESR1 Ser10Ser*	2077647						
TT		26	28.6	335	24.9	1.00	1.00
TC		36	39.6	642	47.7	0.71 (0.42, 1.20)	0.90 (0.46, 1.77)
CC		27	29.7	304	22.6	1.14 (0.65, 1.99)	1.88 (0.94, 3.75)
							
TC/CC		63	69.2	946	68.8	0.85 (0.53, 1.37)	1.21 (0.65, 2.24)
Missing		2	2.2	66	4.8		
							
*ESR1 Ala87Ala*	746432						
GG		67	73.6	1077	80.0	1.00	1.00
GC		15	16.5	181	13.4	1.32 (0.74, 2.36)	1.24 (0.61, 2.51)
CC		1	1.1	7	0.5	2.30 (0.28, 18.95)	3.34 (0.38, 29.18)
*p-for-trend*						0.3	0.3
							
GC/CC		16	17.6	188	14.0	1.36 (0.77, 2.39)	1.31 (0.66, 2.60)
Missing		8	8.8	82	6.1		
							
*ESR1 IVS6+52*	2273206						
GG		69	75.8	1060	78.7	1.00	1.00
GT		13	14.3	229	17.0	0.86 (0.48, 1.59)	0.71 (0.33, 1.53)
TT		3	3.3	10	0.7	4.79 (1.29, 17.88)	5.31 (1.04, 27.28)
							
GT/TT		16	17.6	239	17.7	1.02 (0.58, 1.79)	0.86 (0.43, 1.73)
Missing		6	6.6	48	3.6		
							
*ESR1 Thr594Thr*	2228480						
AA		2	2.2	41	3.0	1.00	1.00
AG		20	22.0	358	26.6	1.14 (0.26, 5.05)	0.88 (0.19, 4.04)
GG		58	63.7	841	62.4	1.42 (0.34, 6.01)	0.86 (0.20, 3.77)
							
AG/GG		78	85.7	1199	89.0	1.34 (0.32, 5.62)	0.87 (0.20, 3.75)
Missing		11	12.1	107	7.9		
							
*ESR1 – 104062C>T*	851982						
CC		38	41.8	470	34.9	1.00	1.00
CT		33	36.3	612	45.4	0.65 (0.40, 1.05)	0.55 (0.30, 0.99)
TT		14	15.4	190	14.1	0.88 (0.47, 1.67)	0.89 (0.43, 1.85)
							
CT/TT		47	51.6	802	59.5	0.70 (0.45, 1.09)	0.63 (0.37, 1.08)
Missing		6	6.6	75	5.6		

**Table 5:** Associations between estrogen receptor-beta (*ESR2*) polymorphisms and breast cancer risk in the BBD cohort

		Cancer		No Cancer			
		(n = 91)		(n = 1347)		All Participants	Post-menopausal Only
	dbSNP rs#	n	%	n	%	Age- Adjusted OR (95% CI)	Age-Adjusted OR (95% CI)
	
*ESR2 38 bp 3' of STP*	4986938						
GG		26	28.6	470	34.9	1.00	1.00
GA		43	47.3	612	45.4	1.27 (0.77, 2.10)	1.46 (0.80, 2.68)
AA		19	20.9	190	14.1	1.83 (0.99, 3.38)	1.71 (0.78, 3.76)
*p-for-trend*						0.06	0.1
							
GA/AA		62	68.1	802	59.5	1.40 (0.88, 2.25)	1.52 (0.85, 2.71)
Missing		3	3.3	75	5.6		
							
*ESR2 5659 bp 3' of STP*	928554						
GG		10	11.0	202	15.0	1.00	1.00
GA		44	48.4	632	46.9	1.41 (0.70, 2.86)	1.04 (0.48, 2.26)
AA		28	30.8	419	31.1	1.38 (0.66, 2.89)	0.88 (0.38, 2.05)
							
GA/AA		72	79.1	1051	78.0	1.40 (0.71, 2.76)	0.98 (0.47, 2.06)
Missing		9	9.9	94	7.0		
							
*ESR2 5696 bp 3' of STP*	none						
AA		75	82.4	1101	81.7	1.00	1.00
AG		12	13.2	180	13.4	0.96 (0.51, 1.81)	0.74 (0.33, 1.67)
GG		1	1.1	4	0.3	3.68 (0.41, 33.37)	--
							
AG/GG		13	14.3	184	13.7	1.02 (0.56, 1.88)	0.73 (0.33, 1.64)
Missing		3	3.3	62	4.6		
							
*ESR2 5772 bp 3' of STP*	8018687						
AA		86	94.5	1133	84.1	1.00	1.00
AG		5	5.5	169	12.5	0.39 (0.16, 0.99)	0.62 (0.24, 1.59)
GG		0	0.0	5	0.4	--	--
							
AG/GG		5	5.5	174	12.9	0.38 (0.15, 0.96)	0.61 (0.24, 1.55)
Missing		0	0.0	40	3.0		
